# Supplementary material for: Comprehensive health risk assessment of microbial indoor air quality in microenvironments
Source: PLoS One. 2022 Feb 25;17(2):e0264226. doi: 10.1371/journal.pone.0264226 (PMC8880710; doi:10.1371/journal.pone.0264226)
Supplement: S1 File — A copy of health proforma and subject consent form used for collecting the health data is provided here. (PDF) [file pone.0264226.s002.pdf]

Comprehensive health risk assessment of microbial indoor air quality in microenvironments of North Delhi

Pradeep Kumar[1], A. B. Singh[2], Rajeev Singh[1]*

1. Department of Environmental Studies, Satyawati College, University of Delhi, Delhi-52, India

2. CSIR- Institute of Genomics and Integrative Biology (IGIB), Delhi University Campus, Delhi-07, India

Email- 10rsingh@gmail.com

Correspondence address- Dr. Rajeev Singh, Department of Environmental Studies, Satyawati College, Ashok Vihar III, Delhi-52, India

The following is the supplementary data:

Questionnaire Form

Form No. SAT/SERB-01

Satyawati College
(University of Delhi)
Ashok Vihar Phase-III, New Delhi-110052

Isolation and Characterization of Bio-contaminant from Indoor Environment in Delhi Residence and their Impact on Human Health

Indoor Air Quality Questionnaire

Name …………………………………………………………………………………………

Father Name: ……………………………………………………………………………… …..

Gender: ……………………………………………………………………………………….

Age: …………………………………………………………………………………………..

D.O.B.: ………………………………………………………………………………………

Profession: …………………………………………………………………………….. ..

Contact No. ……………………………………………………………………….

Email …………………………………………………………………………………… . .

Address. . . . .………………………………………………………………

Enrollment no. ……………………………………………………………………………….

Note: For subject less than 18 years old, this form can be filled by parent

1. How long have you been living in this house?

☐ 0 - 5 Years ☐ 5-10 Years
☐ 10-20 Years ☐ 20- 40 Years
☐ More than 40 Years

2. Date/Year, since you have been living in this house?

☐☐ ☐☐ ☐☐☐☐

3. Approximately how many hours do you spend in this house?

☐ 6 Hours ☐ 12 Hours ☐ More than 12 hours

4. How would you rate the indoor air quality at this building?

☐ Good ☐ Average ☐ Poor

A. Symptoms related to Respiratory Health:

Since you have been living in this house, have you ever been diagnosed with any of the following within last 6 months? Please answer Y for yes and N for no in the box.

5. Do you usually feel Shortness of breath while living in your residential area? ☐

6.Have you ever suffered from Emphysema in last six month? ☐

7.Have you ever suffered from Asthma in last six month? ☐

8.Do you often develop allergic reaction such as itching, rashes etc.? ☐

9.Have you ever got up in night due to coughing or in morning in six months? ☐

10. Do you usually feel Nasal congestion? ☐

B. Symptoms related to General Health:

11. Do you regularly feel unusual thirst (high or low)? ☐

12. Do you usually feel Chapped lips? ☐

13. Have you suffered from Multiple colds (more than four)? ☐

14. Have you ever suffered from Migraines in last six months? ☐

15. Have you ever suffered from Headaches (at least 2/month)? ☐

16. Do you usually feel that your eyes are either burning or irritating? ☐

17. Do you often feel Sneezing attacks? ☐

18. Do you often feel Running nose? ☐

19. Are your symptoms:

☐ Year round ☐ Seasonal ☐ Both

20. If seasonal During which season, do you usually have the above symptoms?

☐ Winter (Dec-Feb) ☐ Spring (Mar-May) ☐ Summer (June-Aug) ☐ Fall (Sept-Nov)

21. At what time of the day your symptoms become worse:

☐ Morning ☐ Afternoon ☐ Night

22. Do your symptoms go away by the morning?

☐ Yes ☐ No ☐ Not applicable

23. Do they go away when you are on vacation?

☐ Yes ☐ No ☐ Not applicable

24. Has there been any evidence of water leaks or visible sign of moisture in your home/ other place?

☐ Yes ☐ No

25. Do you have idea what is reason of symptoms in your residential area?

……………………………………………………………………………………………….

26. Can you offer any comments or observations that may be helpful in improving the environmental condition of your residential area?

…………………………………………………………………………………………………

Signature of Subject/Parent Signature of Research Staff
